# Implementing a multidisciplinary approach for older adults with Cancer: geriatric oncology in practice

**DOI:** 10.1186/s12877-020-01625-5

**Published:** 2020-07-06

**Authors:** Carolyn J. Presley, Jessica L. Krok-Schoen, Sarah A. Wall, Anne M. Noonan, Desiree C. Jones, Edmund Folefac, Nicole Williams, Janine Overcash, Ashley E. Rosko

**Affiliations:** 1 Division of Medical Oncology, Department of Internal Medicine, The Ohio State University, Columbus, OH USA; 2 The James Cancer Hospital/Solove Research Institute, Columbus, USA; 3 Division of Medical Dietetics and Health Sciences, School of Health and Rehabilitation Sciences, The Ohio State University, Columbus, OH USA; 4 Division of Hematology, Department of Internal Medicine, The Ohio State University, Columbus, OH USA; 5 The College of Nursing, The Ohio State University, Columbus, OH USA; 6 A345 Starling Loving Hall, 320 W. 10th Ave, Columbus, OH 43210 USA

**Keywords:** Geriatric assessment, Older adults, Geriatric oncology, Multidisciplinary care

## Abstract

**Background:**

Evidence-based practice in geriatric oncology is growing, and national initiatives have focused on expanding cancer care and research to improve health outcomes for older adults. However, there are still gaps between knowledge and practice for older adults with cancer.

**Main text:**

Here we provide a detailed methodology of geriatric oncology care delivery within a single institution. The Cancer and Aging Resiliency (CARE) clinic is a multidisciplinary approach for implementing geriatric-driven health care for older adults with cancer. The CARE clinic was developed as a direct response to recommendations targeting key multifactorial geriatric health conditions (e.g. falls, nutritional deficits, sensory loss, cognitive impairment, frailty, multiple chronic conditions, and functional status). The multidisciplinary team assesses and delivers a comprehensive set of recommendations, all in one clinic visit, to minimize burden on the patient and the caregiver. The CARE clinic consultative model is a novel approach integrating cancer subspecialties with geriatric oncology healthcare delivery.

**Conclusions:**

Older adults with cancer have unique needs that are independent of routine oncology care. The CARE clinic model provides specific assessments and interventions to improve health outcomes among older adults with cancer.

## Background

Older adults constitute the fastest growing demographic in the U.S. population and are the majority of patients with cancer. By 2030, 70% of all individuals diagnosed with cancer will be above the age of 65 years. However standard hematology/oncology clinical practices are not well adapted to address the unique needs of older adults with cancer [1]. Aging is associated with changes in physiologic function that are important to consider in treatment decisions for older adults with cancer. For example, with age, renal function can decline [2], while the prevalence of anemia is increased [3]. Both of these are risk factors for chemotherapy-related toxicity in patients with cancer. Multiple chronic conditions and polypharmacy can also result in higher levels of treatment toxicity and reduced survival for older adults with cancer [4]. Multifactorial geriatric health conditions such as cognitive impairment, falls, and frailty can also impact patient outcomes but are not routinely assessed or intervened upon in routine cancer care [5]. Multiple organizations including the American Society of Clinical Oncology (ASCO) [6], the International Society of Geriatric Oncology (SIOG) [7], and the National Comprehensive Cancer Network (NCCN) [8], all recommend the inclusion of geriatric principles into cancer care for older adults. Yet practical limitations in healthcare delivery of both geriatric assessments and interventions exist. Therefore, there is a need for innovative health care models designed to harmonize geriatric principles with oncology care with the creation of a multidisciplinary geriatric oncology clinic. Here we outline a detailed description of our geriatric oncology practice, barriers, and practical solutions to implementing a multi-disciplinary care model.

## Main text

Facets of a multidisciplinary team can be highly variable and often consist of physicians (geriatricians, geriatric oncologists, oncologists) conducting a geriatric assessment in concert with other members of an integrated team. We have contrasted our approach to the multi-disciplinary care team model with collated data on other multi-disciplinary clinics nationally through published literature, website review, and personal communications identifying services and resources that are available for older adults with cancer **(**Table 1**).** It is probable that other multi-disciplinary models and clinics exist or/ are in development nationally the dearth of literature around geriatric oncology health care delivery. To date, few centers have published their approach to geriatric oncology practice and therefore, we report on our approach of integrating national recommendations [8] with routine oncology care.

**Table 1:** Multi-disciplinary Clinics in Geriatric Oncology in the United States

Clinic and Location	Components of a multidisciplinary clinic^a^										
	Geriatrics	Oncology	Nursing	Pharmacy	Psycho-social Support^b^	Cognitive/ Psychology/ Psychiatry	Speech Language Pathology	PT	OT	Nutrition	Palliative
Cancer and Aging Resiliency (CARE) Clinic, Columbus, OH	no	**YES**	**YES**	**YES**	**YES**	**YES**	no	**YES**	no	**YES**	no
Memorial Sloan Kettering Cancer Center, New York, NY [9]	**YES**	**YES**	**YES**	**YES**	**YES**	**YES**	no	**YES**	**YES**	**YES**	**YES**
Specialized Oncology Care & Research in the Elderly (SOCARE) Geriatric Oncology Clinic, Wilmot Cancer Institute, Rochester, NY	**YES**	**YES**	**YES**	**YES**	**YES**	**YES**	no	**YES**	**YES**	**YES**	**YES**
Comprehensive Oncology Program for Elders (COPE), Roger Williams Cancer Center, Providence, RI	**YES**	**YES**	**YES**	**YES**	**YES**	no	no	**YES**	**YES**	**YES**	**YES**
Geriatric Oncology Program, Tate Cancer Center, Baltimore, MD	no	**YES**	**YES**	**YES**	**YES**	no	no	**YES**	**YES**	**YES**	**YES**
Center for Cancer and Aging, City of Hope, Duarte, CA	**YES**	**YES**	**YES**	**YES**	**YES**	no	no	**YES**	**YES**	**YES**	no
Senior Adult Oncology Center, Sidney Kimmel Cancer Center, Baltimore, MD [10]	**YES**	**YES**	**YES**	**YES**	**YES**	no	no	no	no	**YES**	**YES**
Geriatric Oncology Program, Lineberger Comprehensive Cancer Center, Chapel Hill, NC	**YES**	**YES**	no	**YES**	**YES**	**YES**	no	**YES**	**YES**	no	no
Levine Cancer Institute, Charlotte, NC	**YES**	**YES**	**YES**	**YES**	**YES**	no	no	no	no	no	**YES**
Senior Adult Oncology Program, Moffitt Cancer Center, Tampa, FL [11]	no	**YES**	**YES**	**YES**	**YES**	no	no	no	no	**YES**	no
Specialized Oncology Care & Research in the Elderly (SOCARE) Clinic, University of Chicago Medicine, Chicago, IL	no	**YES**	**YES**	no	**YES**	no	no	no	no	no	**YES**
Living Well Program, Abramson Cancer Center at Pennsylvania Hospital, Philadelphia, PA [12]	no	**YES**	**YES**	no	**YES**	**YES**	no	no	no	no	no
Geriatric Oncology Clinic, Cleveland Clinic Taussig Cancer Center, Cleveland, OH	**YES**	no	**YES**	**YES**	**YES**	no	no	**YES**	no	no	**YES**
John Theurer Cancer Center, Hackensack University, New Jersey	**YES**	no	**YES**	no	**YES**	no	no	no	no	no	**YES**

Data regarding multidisciplinary care teams was collated via published literature, website review, and personal communications ^a^Based on personal communication with clinic leaders and/or directors. ^b^Can include social workers, patient navigators, health advocates, or chaplains. *PT* Physical Therapy, *OT* Occupational Therapy. Programs are not static and may have changed since the time of this publication. We recommend contacting the programs directly for the most up-to-date services available

**Fig. 1:**
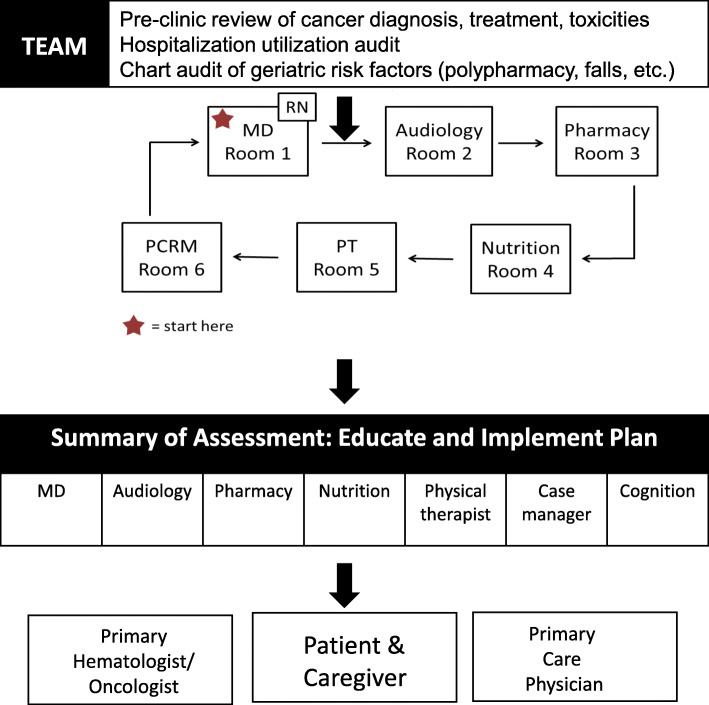
Structure of Workflow in OSUCCC CARE Clinic: Patients are roomed simultaneously and providers rotate to see patients in a “round-robin” fashion. Providers compile a summary of the patients’ needs to create individualized care plans, which are shared with the patient and caregiver at the end of the visit

We created and implemented a multidisciplinary consultative clinic for older adults with cancer branded The Cancer and Aging Resiliency (CARE) clinic. The clinic is a consultative model in which patients are seen for a ‘one-time’ visit where geriatric deficits are assessed and interventions are prescribed; ongoing patient needs are fulfilled through the primary oncology office. The CARE clinic model is comprised of a seven member clinical team, including specialists in the following domains: hematology/oncology, pharmacy, audiology, psychosocial assessment, cognitive evaluation, physical functioning, and nutrition. Patients are assessed in a standardized approach, with each of the seven team members performing targeted assessments to capture age-related risk factors, while eliminating redundancy from provider to provider (see Table 2 for complete list of assessments performed by each provider). At the end of the visit, the team reviews the compiled recommendations with the patient and/or caregiver to summarize the assessment results, appropriate interventions, and educational materials. Clinical summaries regarding the geriatric assessment, strengths, deficits, risk factors, interventions, education and cumulative frailty scores are communicated to the primary oncologist and primary care physician **(**Fig. 1**)**. This is a distinctly different model than the Cancer and Aging Resilience Evaluation developed at University of Alabama-Birmingham, which is a survey-based evaluation, and not a clinic model [18]. The innovation of the CARE clinic model is that it allows multiple services to be centralized for the patient and caregiver, while providing a comprehensive care plan optimizing age-related factors in the context of individual cancer care. The seven facets of our multidisciplinary team are described below, including the logistical approaches used by each team member to provide comprehensive care for older adults with cancer.

**Table 2:** Cancer and Aging Resiliency Clinic Team and Assessment Parameters

	Physician Assessments
Physican	Longevity – SEER^a^ Life Expectancy by gender
	Depression and anxiety – Your Feelings/LASA
	Chemotherapy toxicity – CARG^b^ Chemotherapy Toxicity Calculator
	Geriatric syndromes – incontinence, insomnia, delirium, falls, pressure ulcers, constipation/diarrhea
	Geriatric assessment metrics – fatigue vs exhaustion
	**Audiology** Assessments
Audiologist	Hearing loss – pure tone air and pure tone bone conduction audiometry
	Ear health - otoscopy
	Speech and word recognition thresholds (social disengagement) – ASHA^c^
	Middle ear conduction - tympanometry
	**Pharmacy Assessments**
Pharmacist	Medication reconciliation – Beers Criteria [13]
	Medication management – review of medication names, use, administration schedule
	Clinically relevant medication issues - Drug therapy problems
	Drug-induced neuropathy or ototoxicity
	Potential drug toxicities and interactions – medication history
	**Physical Therapy Assessments**
Physical Therapist	Mobility (fall risk) – Timed Up and Go [14, 15]
	Higher-level balance and postural stability (fall risk) – Functional Gait Assessment
	Functional lower limb strength (fall risk) – 5X sit to stand
	Ambulatory assistive device (fall risk) – grab bars, chairs, rollator, cane, etc.
	Pain – active range of motion/passive range of motion
	**Case Management Assessments**
Nurse Case Manager	Financial toxicity - socioeconomic evaluation
	Independence – ADLs^d^ and IADLs^d^
	Social isolation – caregiver and social support
	Coping – psychosocial referral
	Preparedness – review of advance directives
	**Nutrition Assessments**
Nutritionist	Malnutrition –mini-nutritional assessment screening [16] and counseling
	Digestive issues – diarrhea/constipation
	Strength and energy – nutritional intake
	Body measurements - anthropometrics
	Nutritional education – information booklets
	**Nursing Assessments**
Nurse	Cognition – Blessed [13] or MoCA^e,^[17]
	Laboratory: abnormalities in vitals, hematologic, or chemistry and liver function tests

^a^SEER: Surveillance Epidemiology End Results; ^b^CARG: Cancer and Aging Research Group; ^c^ASHA: American Speech-Language-Hearing Association; ^d^(I)ADL: (Instrumental) Activities of Daily Living; ^e^MoCA: Montreal Cognitive Assessment

The CARE clinic is led by a hematology/oncology physician who reviews and summarizes each patient’s relevant oncologic history (date of diagnosis, stage, treatment plan, chemotherapy, surgical history, radiation exposure). Review of comorbidities, relevant hospitalizations or emergency room use are also noted. The primary objective for the physician is to identify patients’ goals-of-care and barriers to quality of life within the context of a cancer diagnosis. Dialogue regarding the influence of the cancer diagnosis, treatment decisions and toxicities on geriatric syndromes and quality of life are addressed with both the patient and caregiver in a shared decision-making process. Multifactorial geriatric health conditions are identified, and interventions are outlined and education is reviewed. Psychosocial well-being is screened by evaluating anxiety, depression, fatigue, and exhaustion.

A dedicated pharmacist within the clinic reviews patients’ prescription and over-the-counter medications (herbs, supplements, and vitamins), as well as their purposes, dosages, schedules, and side effects. Individual medication lists are reconciled for accuracy, compliance, side effects, and effectiveness with the patient, caregiver, and outpatient pharmacy. Potential therapeutic duplications, drug-drug interactions, or medication inappropriateness are addressed according to the Beers criteria [19]. The CARE pharmacist documents high-risk medications and any medication adjustments made by the CARE physician during the visit. De-prescribing rather than suggesting new prescriptions constitutes the majority of medication recommendations.

The CARE clinic audiologist evaluates each patient in a sound booth located within the clinic. Patients’ otologic history and symptoms are evaluated with a combination of pure tone measurements and speech hearing acuity testing. Characteristics of hearing loss such as the degree of loss, hearing asymmetry, and loss due to medical condition are captured. The audiologist works with patients to identify early indicators of progressive noise-induced hearing loss and advises patients on the use of hearing protection and hearing assistive devices, established to improve health-related quality of life [20].

The clinic’s Patient Care Resource Manager (PCRM) is a nurse patient navigator that functions within the multidisciplinary team as an advocate, educator, and resource for patients. The PCRM assesses function in terms of activities of daily living (ADL) and instrumental ADLs (IADL), which are associated with oncology treatment outcomes [16]. Functional status is a key patient-centered outcome documented in the CARE clinic, as low IADL scores are associated with functional decline after one cycle of chemotherapy [21]. The PCRM also promotes self-care management and prevention with instruction in understanding and managing patients’ diagnoses, treatment, symptoms and clinical trial processes. The PCRM also navigates community resources tailored to meet individual older adult needs (e.g. senior services, insurance coverage, co-pay, transportation, home safety, caregiver concerns, coping).

The CARE clinic nurse evaluates patients using the Blessed Orientation Memory Concentration Test (BOMC) to identify mild, moderate or severe cognitive impairment [13], or the Montreal Cognitive Assessment (MoCA) to evaluate mild cognitive impairment [17]. The clinic nurse has received specific training in administering these cognitive tools. This information is valuable for decision-making capacity, risk for delirium, and life expectancy for both the clinician and patient [22]. Education and resources are provided for patients and caregivers with known cognitive impairments. Complete neuropsychological testing is done by consultation if needed.

The CARE physical therapist (PT) meets with all patients to assess mobility (e.g. range of motion, posture, weight transfer) and objective measures of functional capacity, such as sit to stand [23], functional gait assessment [24], timed up and go [14]. The PT develops a plan using treatment practices to promote movement, reduce pain, restore function, and prevent disability. The PT educates patients how to prevent or manage any physical deconditioning, prevent fall risk and assesses the need for durable medical equipment.

The certified dietician discusses current diet/intake, oral supplements, appetite, caloric need and expenditure, and any barriers to oral intake (e.g. swallowing difficulty) with all patients. Anthropometrics are obtained and recorded. The dietician also administers the mini nutritional assessment [25] where deficits are predictive of an early mortality in an oncology population. Nutritional interventions include nutrient recommendations (calorie, protein, fluid), symptom management (e.g. dysgeusia, mucositis), oral nutritional supplements or appetite stimulants in concert with the prescribing physician. The goal of a nutritional plan is to preserve lean body mass, maintain strength, aid in recovery and healing, and to prevent or reverse nutritional deficits.

Our approach to a consultative multidisciplinary CARE model was to fulfill a need for aging adults with cancer. The CARE clinic model has evolved since conception, the clinic has grown in capacity to care for patients with all cancer types, at any stage. The clinic was initially targeted for octogenarians with hematologic malignancy. This patient population was selected due to vulnerabilities and an inherent need to address multifactorial geriatric health conditions. Over three years, the clinic was expanded to include patients with all malignancies, at any stage of their illness (newly diagnosed, active treatment, previously treated) in addition our clinic now includes Geriatric Oncologists. Notably, there is not a specific age-threshold for consultation; rather, the CARE clinic is designed to address the unique needs of aging with cancer, as the patient and provider deem appropriate.. There is significant heterogeneity in aging. For example, a 60 year old with significant comorbidities and frailty may be “older” and at higher-risk for poorer cancer outcomes as compared to a 75 year old who is still playing tennis and regularly socializing and/or volunteering within their community. For this reason, the CARE clinic model focuses on physiologic rather than chronologic age.

The creation of the multi-disciplinary team model was not a new concept to the institution, but had previously consisted of physicians with different expertise (i.e. surgical oncologist partnered with a medical oncologist). The creation of a multi-disciplinary team with providers from many different departments required a project team and several months of planning to execute a clinic that was interdisciplinary and resourced appropriately. The final disciplines (nursing, case management, nutrition, physical therapy, pharmacy, audiology, physician hematology/oncology) were selected based on the domains of geriatric assessment and clinical time commitment. During the planning phase, many disciplines were approached that were not included in the clinic due to time constraints of the clinic and/or available resources (e.g. integrative oncology, occupational therapy (OT), social work, ophthalmology, dentistry). These additional disciplines are still accessible on a referral basis based on the CARE clinic assessment.

The initial planning of the clinic required commitment and investment from institutional leadership including hospital administration, support of clinical physician faculty from the College of Medicine, and department/divisional support. The planning phases included managerial teams dedicated to streamlining a complex need into an organized service line including allocation of staff with department managerial approvals, creation of memorandum of understanding (MOUs) for staff time commitment, creation of unique templates within the electronic medical record, formation of standardized patient note templates, financial analysis and billing checks, insurance inquires, formatting to allow multiple providers to access a single patient encounter, marketing tools and space allocation. Some initial challenges to overcome included system limitations, consistent staffing, and referral building. Reimbursement is billed separately by each discipline and issues with insurers (Medicare, Medicaid, or private) have occurred in < 1% of all patients evaluated. The CARE model need and purpose required dissemination and education to staff from scheduling, nursing, physicians, patients, and caregivers. Creating the infrastructure to support a Geriatric Oncology clinic required planning, investment, education, and ultimately resulted in a sustainable, novel, clinic to support the community of aging adults with cancer.

The impact of aging for older adults with cancer requires a unique approach that addresses both health factors related to aging and the disease pathogenesis. Therefore, in developing a coordinated care plan, the CARE clinic model considers specific cancer subtypes. We outline specific needs of cancer subspecialties for older adults at the CARE clinic, including hematologic malignancies, as well as lung, gastrointestinal, and breast cancers.

Understanding the impact of aging for older adults with blood cancer requires a unique approach that both addresses the disease pathogenesis and health factors related to aging particularly as it applies to hematopoietic stem cell transplant (HSCT). In terms of treatment for hematologic malignancies, numeric age alone should not be a contraindication to HSCT. The expanded use of alternative stem cell donors (unrelated/haploidentical) [26] and reduced intensity conditioning (RIC) regimens have resulted in increased tolerability of HSCT and similar non-relapse mortality, relapse, disease-free survival, or overall survival (OS) [27]. In allogeneic transplant, GA variables predict OS; specifically, limitations in IADL, slow gait speed, comorbidities, and low mental health scores are all factors shown to be significantly associated with inferior OS [28]. A standard pre-transplant assessment using performance status alone has been shown to not identify frailty in 25% and pre-frailty in 58% of those with “good” health [29]. Factors that enhance transplant-related recovery and interventions to mitigate transplant morbidity are under study within the CARE clinic. For example, low physical pre-transplant function and weight loss are associated with longer transplant hospitalizations [30]. The CARE clinic assesses older adult transplant candidates as well as younger patients for whom the referring provider has specific concerns. The CARE clinic uses a standardized GA, Rockwood’s clinical frailty scale [31], and the 1-year overall Center for International Blood and Marrow Transplant Research survival calculator [32, 33] The CARE clinic’s primary goal in evaluating transplant candidates is to identify deficits and mitigate risk factors to improve tolerability of the transplant.

Lung cancer is particularly challenging for older adults, as it carries a high physical and emotional symptom burden. While there are many new treatments available, clinical trials predominantly sample younger adults, thereby limiting generalizability of outcomes to older adults particularly regarding new treatments [34]. This is particularly relevant in non-small cell lung cancer (NSCLC), where the new standard of care for first-line treatment is now a combination of chemotherapy plus immunotherapy [35]. A lack of clinical trial evidence regarding important clinical outcomes, such as treatment toxicity, disease response, and functional status among older adults receiving lung cancer treatment perpetuates uncertainty for clinicians, patients, and their families [36]. Geriatric-specific assessments [37, 38] have been developed as management tools, which can help guide treatment for older adults with lung cancer, specifically in reducing toxicity exposure while not sacrificing improvement in overall survival. At the CARE clinic, our approach employs GA assessments to identify multifactorial geriatric health conditions and interventions are personalized for patients with lung cancer based on identified deficits. Specific attention to maintaining functional status and preventing functional decline is an active area of research in this area and across malignancies [39].

Gastrointestinal (GI) cancers represent unique challenges for older adults, as they often require multimodality treatment including chemotherapy, radiation therapy and surgery. Determination of fitness for surgery and recovery post-surgery are frequently impacted by comorbidities. Complications of surgery may make nutritional recovery more challenging include pancreatic exocrine insufficiency leading to malabsorption and delayed gastric emptying, which may impair appetite and lead to reduced oral intake. Post-operatively, physicians also need to be cognizant of the increased sensitivity of older adults to side effects from opioid use and the risk for delirium [40]. The GA may uncover risk factors that may predispose a patient to worse surgical outcomes and may help to identify issues such as cancer-associated cachexia and sarcopenia or poor functional status. Importantly, this may trigger a referral for prehab prior to embarking on a major surgical procedure such as a Whipple procedure [41].

Almost one-half of breast cancer diagnoses occur in women age 65 years and older [42]. Although older women are more likely to be treated with lower doses of chemotherapy, studies have shown that older women who are in good health tolerate chemotherapy as well as younger patients [43]. A recent phase II study found that a GA-based risk score predicted treatment toxicity in older adults with metastatic breast cancer who received chemotherapy treatment [44]. At the Breast CARE Clinic, each patient is consented and followed longitudinally with a complete geriatric assessment, focusing on all of the domains described in the CARE clinic model, with the exception of audiology. Identifying gaps and dynamics of geriatric deficits among patients with breast cancer will help identify where clinical outcomes can be improved.

## Conclusions

Recognition of the current evidence in normative aging, multifactorial geriatric health conditions, wellness, and prevention are important aspects of nursing best practices [45]. Given the complexities of geriatric care, providing competent geriatric nursing education is critical to management of care in older adults with cancer. Conducting screening using the GA to identify limitations that may potentially impact cancer care is an important role of primary care and advanced practice nurses [46]. At the CARE clinic, nurses work with the oncology team to develop management strategies intended to enhance functional status and address untreated comorbidities. Nursing teams are trained in Geriatric specific needs (e.g. cognitive assessment, elder abuse) to provide best practice care to older adults with cancer and is a major priority of the CARE clinic.

As cancer survivors live longer, evaluating long-term adverse effects is imperative [47]. These include physical and psychological issues including, fatigue, pain, osteoporosis, cardiac toxicity, weight and nutritional changes, cognitive changes, depression, anxiety, and neuropathy, among others [48]. Furthermore diminished social and economic resources may also impact the survivorship experience [49]. This myriad of unmet needs and complexities, coupled with the reduction in services available for older cancer survivors post-treatment, warrant a more robust provision of survivorship follow-up care [50, 51]. Addressing survivorship among older adults requires a comprehensive approach considering recommended follow-up care, managing multi-morbidity and medications, deciphering between age- or cancer-related physical and mental symptoms, and coordinating care from multiple physicians [52]. Survivorship care plans (SCPs) may improve existing and potential survivorship issues experienced by cancer survivors after cancer treatment. SCPs include key information regarding cancer and treatment, potential late or long-term adverse events, surveillance, and health lifestyle recommendations, and identification of providers who will coordinate care [53]. SCPs should include tailored information to address the needs of older adults including modifiable health behaviors (e.g. diet, exercise), polypharmacy, comorbidities, and social support [54, 55]. Strategies include the utilization of geriatric assessment to outline health concerns, care coordination models to outline the responsibilities of various providers for comorbid conditions, and partnering with caregivers in the care delivery. The CARE clinic at OSUCCC is developing and implementing SCPs into routine cancer care for older adults.

The practical approach to Geriatric Oncology health care delivery across institutions is not well described. Here we identify the specific members and role of our multi-disciplinary team, provide detailed assessment tools used within the clinic, practical interventions, and unique needs of individual cancer subspecialties. Unaddressed geriatric health conditions can result in costly and uncoordinated care, worsening the quality of life for older adults with cancer and their caregivers. The CARE Clinic is a multidisciplinary approach to address the unmet needs of older adults with cancer designed specifically to address risk factors, implement interventions, and streamline care in concert with routine oncology care.

## Data Availability

Not applicable.

## Abbreviations

ADL: Activities of daily living
BOMC: Blessed Orientation Memory Concentration
CARE: Cancer and Aging REsiliency
GA: Geriatric Assessment
GI: Gastrointestinal
HSCT: Hematopoietic stem cell transplant
IADL: Instrumental activities of daily living
MoCA: Montreal Cognitive Assessment
MOU: Memorandum of understanding
NSCLC: Non-small cell lung cancer
OS: Overall survival
OSUCCC: The Ohio State University Comprehensive Cancer Center
OT: Occupational therapy
PCRM: Patient Care Resource Manager
PT: Physical therapist
RIC: Reduced intensity conditioning
RN: Registered nurse
SCP: Survivorship care plan
